# Enhancing Reproductive Performance of Freshwater Finfish Species through Dietary Lipids

**DOI:** 10.1155/2022/7138012

**Published:** 2022-11-28

**Authors:** Donald Torsabo, Sairatul Dahlianis Ishak, Noordiyana Mat Noordin, Ivan Chong Chu Koh, Muhammad Yazed Abduh, Benedict Terkula Iber, Meng-Kiat Kuah, Ambok Bolong Abol-Munafi

**Affiliations:** ^1^Higher Institution Centre of Excellence (HICoE), Institute of Tropical Aquaculture and Fisheries, Universiti Malaysia Terengganu, 21030 Kuala Nerus, Terengganu, Malaysia; ^2^Department of Fisheries and Aquaculture, Federal University of Agriculture Makurdi, Makurdi, Benue State, Nigeria; ^3^Faculty of Fisheries and Food Sciences, Universiti Malaysia Terengganu, 21030 Kuala Nerus, Terengganu, Malaysia; ^4^Lab-Ind Resource Sdn Bhd, 48300 Bandar Bukit Beruntung, Selangor, Malaysia

## Abstract

Dietary lipid manipulation in the feed of commercially cultured finfish is used not only to improve production and culture but also to enhance their reproductive performances. The inclusion of lipid in broodstock diet positively affects growth, immunological responses, gonadogenesis, and larval survival. In this review, existing literature on the importance of freshwater finfish species to aquaculture and the inclusion of dietary lipid compounds in freshwater fish feed to accelerate the reproduction rate is being summarized and discussed. Although lipid compounds have been confirmed to improve reproductive performance, only a few members of the most economically important species have reaped benefits from quantitative and qualitative lipid studies. There is a knowledge gap on the effective inclusion and utilization of dietary lipids on gonad maturation, fecundity, fertilization, egg morphology, hatching rate, and consequently, larval quality contributing to the survival and good performance of freshwater fish culture. This review provides a baseline for potential future research for optimizing dietary lipid inclusion in freshwater broodstock diets.

## 1. Introduction

Fish go through various developmental processes such as gametogenesis, spawning, hatching, and larval development, and the success of these processes heavily relied on good nutrition provided to the broodstock [1–4]. Broodstock nutrition is essential in the aquaculture sector and calls for optimum conditions, such as giving the fish ideal ambient conditions and a diet that maximizes reproductive function to produce high-quality offspring [5, 6]. The feeding regime, which comprises the nutritional status of the larvae, the length of feeding, and the effectiveness of maternal nutrition, presents difficulties and problems during the early phases of finfish larval rearing [7, 8]. Fish broodstock with dietary nutrient imbalances have severe reproductive effects on the success of egg fertilization because it impairs vitellogenesis, oocyte maturation, and spawning. Fortunately, manipulation of nutrients in diet of broodstock has been shown to increase nutrient efficiency, resulting in robust and healthy descendants [7, 9, 10].

Lipids and their constituent fatty acids play major roles in the provision of metabolic energy in fish including growth and reproduction [11]. Lipids of particular nutritional importance in fish are essential fatty acids (EFA) because they play a strategic role in fish embryonic development. Long-chain polyunsaturated fatty acids (LC-PUFAs) such as docosahexaenoic acid (DHA:22:6n-3) and eicosapentaenoic acid (EPA:20:5n-3) are important in the broodstock diet for their role in egg and larval development [12]. In addition, focus has also been given on arachidonic acid (ARA:20:4n-6) because of its role in the production of more robust eicosanoid which is involved in diverse physiological functions, including reproduction and egg development [13]. Like other animals, fish obtain these EFAs from their diet because they are incapable of synthesizing them within their body system. Different fish species at different life stages have specific requirements for these EFAs and therefore must be provided in their diets to meet the specific body functions.

Propagation and rearing of freshwater fish for human consumption and other economic benefits have expanded the aquaculture industry at a remarkable rate. In 2004, freshwater aquaculture production was only 25.6 million tonnes [14]. However, in 2018, over sixty percent of current world production are from freshwater species at a total of 51.3 million tonnes [15]. Other than the global demand for a sustainable protein source, the ongoing growth in the volume and supply chain of freshwater species aquaculture is attributable to increased resource exploitation and intensification of what was once a household-managed or medium-scale commercial activity [16]. The continuity and sustenance of the high volume of freshwater aquaculture production depend on several factors such as environmental, nutritional, and availability of quality seed [17, 18]. The scarcity of seeds is among significant impediments to the development of freshwater aquaculture. Production is entirely reliant on the extraction of wild population broodstock and/or its seeds and inducement of broodstock with purified gonadotropin hormone such as human chorionic gonadotropin (HCG) for many farmed species [19, 20]. The application of hormone during artificial breeding does not guarantee success in breeding induction if the broodstocks are in a poor nutritional state [1]. Full control of broodstock sexual maturity from all aspects will influence their spawning performance, an eminent factor in flourishing freshwater aquaculture production.

The aim of this article is to provide a comprehensive insight into the lipid nutrition in freshwater fish broodstock diets. The subsequent goal is to recognize knowledge gaps and research needs in the use of dietary lipids to improve reproductive performance in freshwater fish species in ensuring the long-term viability of the rapidly growing aquaculture industry.

## 2. Freshwater Fishes in Aquaculture

**Figure 1:**
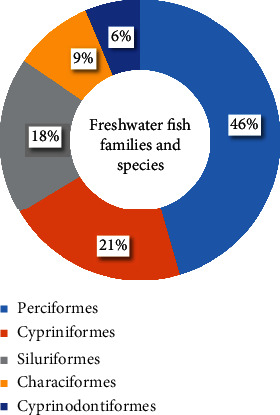
Composition of freshwater fish families and species by abundance as reported by Vander Sleen and Albert [23].

Freshwater fishes are species that spend the majority of their lives or a significant portion of their lives in freshwater inland or brackish bodies of water. All stenohaline, such as carps and cichlids, and euryhaline, such as salmon, eels, and several species of elasmobranchs, are included in this definition [21]. Database showed that more than 33500 species of fishes described to date, with around 18,000 fish species that inhabit freshwater rivers, lakes, and wetlands, and hundreds of new species are being discovered every year. [22, 23]. Surprisingly, only five taxonomic orders account for around 85% of the diversity of freshwater fish worldwide (Figure 1).

Farmed freshwater fish have contributed immensely to aquaculture production over the years. Cyprinids and carps are currently leading in the chart of aquaculture fish production, contributing to total fish production at approximately 53.1%, followed by miscellaneous freshwater species (19.5%), tilapia and other cichlids (11.0%), diadromous salmonids (6.5%), and lastly the miscellaneous coastal fishes with 2.8% [24].

Despite the immense contribution of freshwater fish species to aquaculture production, bottlenecks delaying the exponential growth of production include inadequate broodstock nutrition which in turn constitute issues of late maturation of broodstock, spawning in captivity, egg production, hatchability of eggs, and larval survival in some species, especially the newly introduced in aquaculture [25, 26]. Many broodstock management studies through nutritional aspects have been focused on the studies of dietary lipids and its contribution in improving the reproductive performance of different freshwater fish species.

## 3. LC-PUFA Biosynthesis Pathway in Freshwater Fishes

**Figure 2:**
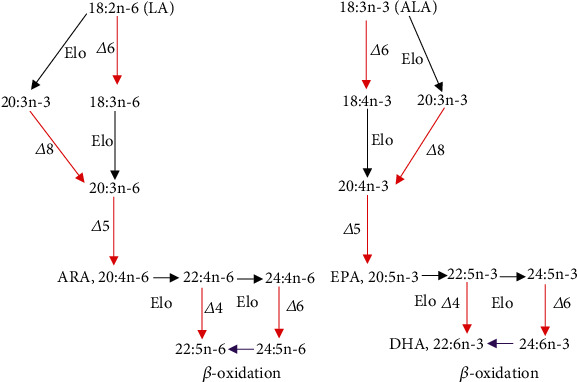
LC-PUFA pathways from the C18 precursors LA, C18: 2n-6 and LNA, C18:3n-3. Red arrows represent fatty acid desaturase while black arrows represent fatty acid elongase. Purple lines represent steps that have not been directly demonstrated in fish. *Δ*8, *Δ*6, *Δ*5, and *Δ*4: fatty acid desaturases; Elo: fatty acid elongases; short: peroxisomal chain shortening [28].

Vertebrates including fish are incapable of de novo synthesis of the two important polyunsaturated fatty acids (PUFA) which are linoleic acid (LA, C18:2n-6) and linolenic acid (LNA, C18:3n-3) [27]. This is due to the absence of the *Δ*12 and *Δ*15 fatty acid desaturase enzymes which are required for the LA and LNA production from oleic acid (C18:1n-9) [28]. LA and LNA are essential as they are precursors to produce long-chain polyunsaturated fatty acids (LC-PUFA), ARA, EPA, and DHA. The *in vivo* conversion of LA and LNA to ARA, EPA, and DHA, respectively, can be made via two pathways through an alternating sequence of desaturation and elongation, both regulated by the desaturase and elongase enzymes [28–31]. These two pathways are as described in Figure 2.

Emphasis in many studies has been given to the production of EPA, DHA, and ARA, as they are the precursors of eicosanoids that are integral for array of cellular activities including membrane integrity, immune response, gene regulation, and renal and neural functions [31]. Prostaglandins, the oxygenated metabolites of ARA and EPA, are key players in the vertebrate development, with ARA being more dominant [32]. Eicosanoids derived from ARA are reported to greatly improve immune functions and survival rate in Japanese eel (*Anguilla japonica*) and rabbitfish (*Siganus rivulatus*) [33, 34]. EPA may also form eicosanoids but has been suggested to be less biologically active than those formed from ARA but yet may competitively interfere with the actions of ARA-formed eicosanoids [35, 36]. DHA has a critical role in neurophysiological functions of fish especially in the brain; thus, its deficiency led to lower stress tolerance [27]. Negative long-term effect on neural tissues has been seen in the early life stages of pikeperch (*Sander lucioperca*) [37], impaired vision for prey capture in larvae of Atlantic bluefin tuna (*Thunnus thynnus*) [38], and also schooling behavior of larvae and juveniles of yellowtail (*Seriola quinqueradiata*) [39].

Freshwater species has demonstrated the ability to desaturate and elongate LA, 18:2n-6 and ALA, 18:3n-3 into LC-PUFA according to their needs [40, 41]. In a study of female broodstock of *Tor tambroides*, DHA content in the muscle of the fish fed with diet low in LC-PUFA increased, illustrating the ability of *T. tambroides* to synthesize DHA de novo [1]. Similar abilities have been also demonstrated in cyprinids Tench (*Tinca tinca*) [25], silver barb (*Barbonymus gonionotus*) [42], Nile tilapia (*Oreochromis niloticus*) [43], and the striped snakehead (*Channa striata*) [44]. Manipulation of dietary fatty acids in these studies may result in increased activities of the fatty acyl desaturation and elongation pathway in isolation [45, 46]. In addition, significant findings have been made in identifying genes involved in fatty acid metabolism at molecular level [47] and enzymes that possess function capacity in biosynthesis of EPA, DHA, and ARA [25] in freshwater species. In comparison, marine fishes exhibit lack of delta-5 desaturase activity characterized by loss of Fads1 gene, consequently, impairing EPA and DHA synthesis [48–50].

## 4. Lipid Sources

Lipid-based ingredients represent the highest energy source in fish diet in comparison to protein and carbohydrate and are important for fish growth and reproduction [51]. In comparison to plant-based lipid, fish-based meal and oils were traditionally preferred in the formulation of fish diets in the past, which is scientifically proven to be nutritionally beneficial [52, 53]. Despite the success of using fish oil in fish feed formulations, global fish oil output is unpredictable, and there is no guarantee of increased production even if sustainable fisheries are managed [54, 55]. The progressive growth experienced in aquaculture production has necessitated the use of alternative lipid sources from terrestrial plants to make available the amount of oil required to sustain aquaculture production worldwide [52, 56, 57]. Crop oils such as soybean, sunflower, and linseed oils have proved to be an efficient source of lipids in the aquaculture of certain freshwater fish species [56, 58]. There is also the development of insect-based oil usage in aquaculture feed such as the black soldier fly (*Hermetia illucens*) oil, as an alternative lipid source [59].

**Figure 3:**
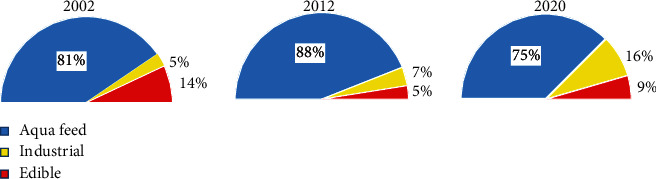
Global estimated use of fish oil in three different sectors (aquaculture feeds, industrial, and edible) in 2002, 2012, and 2020 [15, 52, 61].

The choice of lipids to be used is largely dependent on the dietary requirement of fish based on species, age, size, and the culture environment [60]. Despite the search for alternative lipid sources in aquaculture feed production, the demand for fish oil has been on the increase [61]. The use of fish oil for aquaculture feed production in 2018 was projected to be about 18 million tonnes higher than the usage of 14 million tonnes recorded in 2014 [15]. The Marine Ingredients Organization (IFFO) reported that about 75% of fish oil produced on yearly basis are used in the aquaculture industries for feed production [15]. The increase in demand for fish oil in the aquaculture feed industry is because fish oil is still the most reliable source of n-3 fatty acids, especially EPA and DHA, and a better ingredient substitute has not yet been found.  Figure 3 shows the estimated use of fish oil in three different sectors throughout the years 2002, 2012, and 2020.

## 5. Lipid and Fatty Acid Requirement during Fish Reproductive Stages

**Figure 4:**
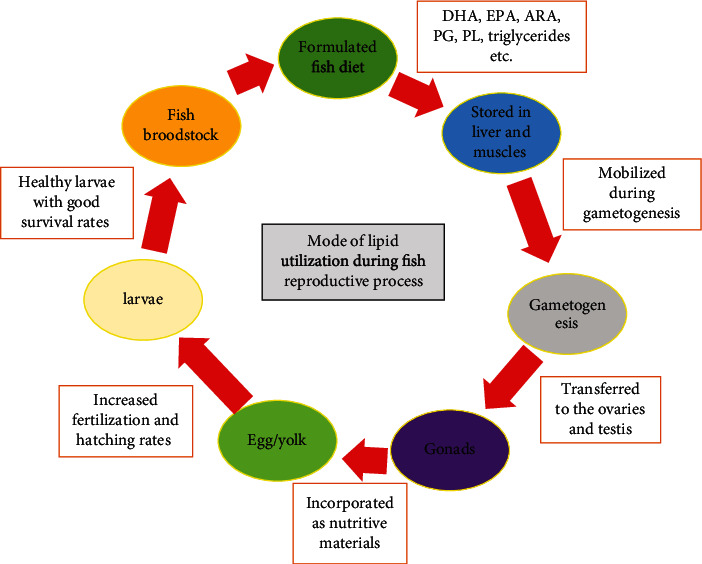
Mode of lipid utilization during fish reproductive processes.

Dietary lipids, which comprise fats and oils (triglycerides), are used by fish as a source of metabolic energy, stress resistance, immune development, hormone precursors, and essential fatty acid (EFA) sources [19, 62]. Lipids and various classes of fatty acids (FA) provide the energy required by broodstock during gonadogenesis and embryogenesis and are regarded as crucial components of cell structures and organelles during organ development [63]. Fatty acid requirements during gonadogenesis and larval development are dictated by the individual levels of each dietary FA and relative proportions of the FA main classes, which can have a significant impact on reproduction success [63–65]. Lipid manipulation in broodstock diets of several fish species has proven to be effective in promoting reproductive performance. Lipid movement from both the liver and muscle of white seabream, *Diplodus sargus*, to its gonads during gonadogenesis [66] and FA composition changes in gonad tissues of rainbow trout, *Oncorhynchus mykiss* broodstock [67], suggest that dietary lipid intake affects broodstock lipid composition and subsequently dictates fish reproductive maturation and gonadogenesis processes. Consistently, an increase of egg size and lipid droplets in the ovary histology and lipid droplets in muscle sections decreased in Chinese sturgeon (*Acipenser sinensis*), which suggests the consumption of muscle energy/lipid reservoir during ovarian development from stage II to IV [68]. In another instance, Chinese sturgeon fed with a higher lipid level diet could develop their ovaries to stage III or IV while they showed constant body weight and decreasing muscle lipid droplets [69]. These suggest that the energy required for ovary development in this fish is of dual source, including exogenous food and endogenous sources (liver, muscle, and visceral fat), as reported in other fish species. Dietary LC-PUFA inclusion in fish feed has been observed to improve egg quality, fertility, and fertilisation success [7]. The mode of lipid utilization during reproductive processes in fish is illustrated in Figure 4.

Significant quantities of lipids are necessary both for the development of female eggs and for male reproductive behaviors, such as increased swimming, parental care, and courtship [70–72]. Lipids provide essential FAs in fish diets, and the EFA requirement of fish can be made sufficient by the supply of the LC-PUFAs of linolenic linoleic series [73]. Dietary lipids are also vital as precursors of steroid hormones and prostaglandins (PGs) which act as a transport pathway for fat-soluble vitamin absorption in fish intestine [73, 74].

The importance of lipids in broodstock diets has been documented in several freshwater fish species such as Nile tilapia, *Oreochromis niloticus*, [7]; striped catfish, *Pangasianodon hypophthalmus* [20]; and common carp, *Cyprinus carpio* [75]. In many instances, studies on the lipid requirement of freshwater fish are conducted to assess their impact on various processes of reproduction such as gametogenesis, fecundity, fertilization, egg morphology, egg hatching rate, larval quality, and survival.

### 5.1. Gametogenesis

Gamete formation requires the processes of generation, motility, multiplication, specialization, and ovulation/spermiation which are identical among fishes [5]. Many fish species in the wild that experience starvation have skipped spawning due to low-fat reserves which demonstrate the significance of an appropriate diet for effective gametogenesis [5]. Fish reproductive activities are controlled by the hypothalamus-pituitary-gonadal (HPG) axis, also known as the reproductive endocrine axis, which has been well studied and characterized [76].

Reproductive activities such as puberty, oogenesis, spermatogenesis, and spawning, including certain cellular processes such as steroidogenesis and sperm maturation, are regulated by the HPG axis [77]. In fish, an adequate supply of energy is crucial for the initiation of reproductive events which begins with the formation and subsequent maturation of gamete. Therefore, the HPG axis depends on the adequate supply of energy during puberty and adulthood for optimum reproductive performance [78].

Lipids are retained in the muscle and liver of fish, mobilized during gametogenesis, transferred to the ovaries from maternal reserves, and introduced into the egg/yolk as nutritional material, which acts as the primary food source for the future embryo [26]. During oocyte maturation, changes in the concentrations and amounts of different lipid groups and FA constituents in various tissues are closely related to both their roles and the fish's reproductive capacity [79]. Dietary lipid inclusion in the right quantity in fish broodstock diet has beneficial effects on the reproductive performance and seed production in fish. Du et al. [80] reported that high lipid level inclusion of fish oil at 10%, 14%, and 18% in the Chinese sturgeon broodstock diets promoted the beginning of puberty and ovary development. Chinese sturgeon fed 10% and 14% lipid had ovary development to stage II with small white eggs of 0.3–0.9 mm in diameter, while those feed 18% lipid recorded stage III of ovary development with a yellow oocyte diameter of 2.0–2.6 mm and grey oocyte diameter of 2.6–3.7 mm. The stagnation in ovary development from stage II to stage III was attributed to insufficient lipid intake and its storage in the body due to inadequate dietary lipid inclusion in the broodstock diet. The promotion of puberty and early gamete formation in both male and female fish has also been described in rabbitfish (*Siganus guttatus*) [7]. Through the introduction of proper broodstock feeding and the combination of adequate lipid sources in fish diets, early maturation of oocytes and seed quality enhancement can be accomplished.

### 5.2. Fecundity

Fecundity is the total production of eggs by different fishes which is by number of eggs per spawn or eggs per total body weight [7]. Fecundity is an essential factor in determining the fish's reproductive performance which is impacted by nutrient intake [20, 81, 82]. Nutritional deficiency of broodstock diets has been reported to have negative effects on the quality and quantity of eggs produced per fish [7]. The increment of lipid levels from 100 g kg^−1^ to 180 g kg^−1^ in broodstock diets for snakehead murrel, *Channa* striata, resulted in a significantly better gonadosomatic index and fecundity [19]. Comparably, the increase of lipid levels from 60 g kg^−1^ to 120 g kg^−1^ in striped catfish, *P. hypophthalmus*, broodstock diets showed improved fecundity[20]. Similarly, 110 g kg^−1^inclusion of lipid in common carp, *Cyprinus carpio*, diets also caused an increase in gonadosomatic index and fecundity [75]. In another study, supplementation of PUFA in diets of common carp *Cyprinus carpio* increases their relative fecundity (1.25) compared to the control (0.69) group fed with a diet devoid of PUFA [83]. Consistently, *Oreochromis karongae* fed 10.17% and 12.09% lipid had the highest absolute fecundity (237.5 ± 6.50 and 271.3 ± 26.19, respectively) as compared with fish fed on 8.28% and 14.05% lipid levels (90.3 ± 46.3 and 143.7 ± 30.8, respectively [82]. The increase in fecundity linked to the increase in lipid levels in the diets of various broodstock is suggested to be correlated with the gradual increase in the dietary EFA content, thus significantly affecting the reproductive performance of fish [7, 81].

### 5.3. Fertilization

Fertilization serves as an important parameter to accurately estimate the quality of egg [84]. An increase in lipid levels from 60 g kg^−1^ to 180 g kg^−1^ in the broodstock diet of striped catfish, *P. hypophthalmus*, resulted in a significantly higher fertilization rate [20]. Better fertilization rate in the eggs of common carp, *C. carpio*, is reported to be at the optimal crude lipid level of 14% [83]. The influence of lipids on the fertilization rate has equally been reported in zebra fish, *Danio rerio* [85]; striped catfish, *P. hypophthalmus* [86]; silver catfish, *Rhamdia quelen* [87]; Siberian sturgeon, *Acipenser baeri* [65]; and fancy koi, *Cyprinus carpio* var. *koi* [88].

### 5.4. Egg Morphology and Hatching Rate

The quality of fish eggs can be quantified based on the fertilization rate and development of successful viable progeny [12]. Factors that influence egg quality include female size, age, nutrition, genetics, origin, and environmental conditions among others [89].

Nutrition has a significant role in progeny development as essential nutrients required are absorbed into the egg at the onset or during vitellogenesis [12]. Lipids have been investigated for their major role in broodstock nutrition with an immense effect on the quality of eggs and larval development [7, 67, 90]. Furthermore, different dietary lipid sources have shown significant effects on egg quality and are reported to have a direct link between the FA contents in broodstock diets and the eggs produced [91]. In a study, European eel broodstock fed diets rich in EFA showed a high number of floating eggs and the egg total lipid content, as well as hatching success [12].

The content of LC-PUFA in broodstock diets directly affects the LC-PUFA content in the early stages of development for eggs and larvae [91, 92]. Similarly, better egg quality and hatching rate in fish have been linked to the presence of dietary LC-PUFA [7]. Consequently, freshwater fish have been classified into three types based on their EFA requirements; these include the rainbow trout type which requires n-3 PUFA like 18:3n-3, while the tilapia type requires n-6 PUFA as EFA and the carp group requires both n-3 and n-6 PUFA [93]. This classification serves as a guide for the inclusion of EFAs and their ratios in feed formulation for freshwater fish species. Furuita et al. [74] investigated the effect of n-3 and n-6 fatty acids in broodstock diet on reproduction and fatty acid composition of broodstock and eggs in the Japanese eel *Anguilla japonica*; results showed that diet containing pollack liver oil (PO) and/or corn oil (CO) and a mixture of PO and CO in ratio 1 : 1 resulted in higher hatching rate in the mixed diet (9.6 ± 9.0) compared to the PO (7.9 ± 14.7) and CO (5.2 ± 4.9) diet alone. This result is an indication that the Japanese eel requires both n-3 and n-6 PUFAs in their diet for improved reproductive performances. A combination of plant and fish oils (i.e., corn oil and pollack oil) is better than plant oil or fish oil alone for the diets, in respect of egg quality and fatty acid composition of broodstock and eggs.

Similarly, the supplementation of n-6 and n-3 PUFAs in the diet of female carp broodstock has been shown to improve gonadal maturation, breeding performance, and spawn recovery. The results in a feeding trial [83] with different sources of oil, fish oil (FO), safflower oil (SO), perilla oil (PO), and a mixture of safflower and fish oil (SO+FO) representing n-6 LC-PUFA and n-3 LC-PUFA showed that the number of matured female carps was the highest in SO+FO (fully bred 100 ± 0.00%), compared to the single supplementation of FO (fully bred 80.00 ± 1.81%), SO (fully bred 78.00 ± 2.25%), and PO (fully bred 76.00 ± 3.08%), and the lowest in control (fully bred 60.00 ± 1.23%). The relative fecundity and fertilization rate also significantly increased in SO+FO, compared to FO, SO, and PO as well as the control (*p* < 0.05). Similar trends were also reported for female carp *Catlacatla* [94] and for male and female carp, *C. catla* [95]. The need for n-3 PUFAs and n-6 PUFAs has been adequately demonstrated in carp species in regard to their reproductive performance enhancement. Although a group of fatty acid requirements has been proven, studies on specific quantitative EFA inclusion has not been given due attention.

In a related development, Sink and Lochmann [96] reported that larval hatching success of the channel catfish, *Ictalurus punctatus*, strongly correlated with individual weight of the egg, total lipid content, n-3 LC-PUFA content, n-3 to n-6 LC-PUFA ratio, and moderate positive correlation with individual egg density. In the same study, a 10% dietary lipid level produced heavier egg masses and significant volume of eggs in spawning than those fed the 4% lipid. Similarly, *C. carpio* fed with 11% lipid levels in their diets have a significantly higher hatching rate [75]. Egg diameter and individual egg density are equally reported to be significantly affected by lipid levels in *C. carpio* [97]; *P. hypophthalmus* [20]; yellowfin sea bream, *Acanthopagrus latus* [8]; and Karonga tilapia, *Oreochromis karongae* [82]. Furthermore, Atlantic halibut, *Hippoglossus hippoglossus*, fed a standard diet containing herring meal and oil and enhanced ARA diets during their two spawning seasons recorded a higher hatching rate (41.0 ± 7.8; 51.0 ± 3.6) in the enhanced ARA group compared to the standard diet (14.0 ± 4.1; 28.0 ± 5.7), respectively [13]. This has demonstrated the active production of eicosanoid by ARA and its involvement in reproductive activities in fish.

### 5.5. Larval Quality and Survival

The quality of larvae and their survival have been linked to the supply of adequate dietary lipids. Larvae of several fish species have underdeveloped mouth parts and intestinal tracts upon hatching and are unable to immediately begin exogenous feeding, consequently obtaining nutrients from endogenous energy reserves from yolk during embryogenesis and then throughout early larval growth before first feeding [51]. It has been reported that European eel broodstock fed EFA-enhanced diet containing different ratios of ARA, DHA, and EPA, with high levels of ARA and DHA with intermediate levels of EPA, shows increased total lipid content of the eggs and improved larval survival [12]. Similarly, increased n-3 LC-PUFA levels in the diet of European perch, *Perca fluviatilis*, broodstocks have been documented to significantly improve larval weight, survival percentage, and osmotic shock tolerance [98]. In the same manner, common carp broodstock fed diet containing 11% lipid recorded the lowest mortality rate of larvae [75].

Total dietary replacement of fish oil that contains n-3 LC-PUFA with vegetable oil that majorly contains n-6 LC-PUFA had negative effects on reproductive performance of rainbow trout *Oncorhynchus mykiss* [99]. In this study, broodstock fed 100% vegetable oil recorded the lowest fertilization rate (81.3 ± 2.3%), compared to brooders fed 80% fish oil and 20% vegetable oil (91.7 ± 1.7%). Survival rates of eyed embryo stage were observed to be highest in brooders fed 50% fish oil and 50% vegetable oil (96.6 ± 5.3) compared to 100% vegetable oil (94.0 ± 1.1). The negative effects of high n-6 LC-PUFA on reproductive performance of rainbow trout might be attributed to the nature of their feeding habit and also their requirement for high n-3 LC- PUFA as their source of EFA to promote reproductive performance and other related metabolic activities [74, 100].  Table 1 shows the effects of various sources and levels of dietary lipids on fish reproductive parameters.

### 5.6. Lipid Metabolism-Related Genes Modulating Fish Reproductive Performance

The lipid metabolic pathways in teleosts; lipolysis, *β*-oxidation, lipogenesis, phospholipid and triacylglycerol syntheses play a critical role in providing metabolic energy sources used for growth, reproduction and other major physiological processes [31]. The liver is the main organ responsible for nutrient metabolic pathways (lipid, protein, and carbohydrates), nutrient storage, enzyme activation, and many vital physiological regulations. Thus, hepatic gene expression studies easily demonstrated effects on sexual hormones and enzymes regulating spermatogenesis and oogenesis [105, 106]. Although investigations using hepatic samples agree that these genes are directly dietary-regulated, practical feeding trials that elucidate nutrient and genomic influence in freshwater finfish reproduction are still far in between. Molecular characterization studies in freshwater species like zebrafish (*Danio rerio*) and yellow catfish (*Pelteobagrus fulvidraco*) have identified the following lipid metabolism-related genes to be regulated during oogenesis and embryogenesis: acetyl-CoA (ACC), carnitine palmitoyltransferase a (CPT1), mitochondrial carnitine palmitoyl transferase I alpha 1a (CPT2), peroxisome proliferator-activated receptor (PPAR), sterol regulatory element binding protein (SREBP), glucose 6-phosphate dehydrogenase (G6PD), 6-phosphogluconate dehydrogenase (6PGD), fatty acid synthase (FAS), lipoprotein lipase (LPL) [47], retinol x receptor (RXR), and liver x receptor (LXR) [107].

This section summarized several expression studies of the genes, enzymes, and transcriptional factors that are modulated by dietary lipid which subsequently influence freshwater finfish reproduction, as shown in Table 2. As previously discussed, freshwater finfish have the genetic capacity for LC-PUFA biosynthesis in which LC-PUFAs are essential in ontogeny especially during embryonic development. Two groups of pivotal genes involved in the LC-PUFA biosynthesis pathway are the fatty acid desaturase (FAD) and elongase (ELOVL) genes. Freshwater fish displayed desaturation activities with bifunctional ∆6/∆5 desaturases in zebrafish (*Danio rerio*) [108] and ∆6 in common carp [45]. Monroig et al. [109] have characterized a FAD2 gene with ∆8 desaturase activity in freshwater fish which complements the *Δ*6 route for the desaturation of C20:3n-3 and C20:2n-6 to C20:4n-3 and C20:3n-6, respectively, hence, proving the efficiency of freshwater fish in LC-PUFA biosynthesis as compared to marine fish. Ishak et al. [110] reported that the FAD6 and ELOVL5 genes regulate vitellogenesis which is a process of nutrient deposition for yolk formation in different stages of zebrafish follicles. Increased FAD gene expression is also shown during larval growth stages indicating its importance during ontogeny for organ development in striped snakehead, *Channa striata* [111]. Choosing the correct source of lipids and LC-PUFA levels also improves female reproductive parameters such as egg fertilization and hatching rate in female swordtail (*Xiphophorus hellerii*) and zebrafish [10, 112].

The gene expression of Apolipoprotein (APO) in ovary and embryos of Chinese sturgeon (*Acipenser sinensis*) indicates the suitability of the APO gene as a marker for endogenous lipid nutrition in the ontogenic study [113]. Sarameh et al. [104] reported that Chinese sturgeon broodstock benefits from kilka fish oil and rapeseed oil mix in diets shown by upregulated APO expression with improved egg fertility and larval survival. Additionally, 18% dietary lipid can increase lipid deposition during vitellogenesis shown by upregulated expressions of fatty acid binding protein (FABP) and lipoprotein lipase (LPL) genes, which then stimulate the development of oocyte follicles to stage IV maturation [68].

The application of -omics studies may provide clearer information to better understand the complex process of freshwater finfish reproduction, both male and female.

## 6. Conclusion and Future Work

This review has made it clear that dietary lipids over the years have shown to be an essential element in the diets of various freshwater fish species to enhance their reproductive performance. Dietary lipids, despite being an integral element in aquaculture nutrition to improve the reproductive performance of freshwater fish species, have not been studied in line with improving their reproductive performance, but rather, the concentration of various researchers has been geared towards sparing proteins and growth performance in aquafeed. Special attention should be given to dietary requirement of broodstock, particularly during different reproductive stages, to guarantee quality offspring. It is also critical to expand research on the numerous fish species, especially on the newly introduced domesticated species, as only a few of the economically significant species have been studied. Studies on the effects of fish oil replacement with plant oil sources in aquaculture feed on the reproductive performance of freshwater fish species are necessary as less attention has been given to this aspect in the past.

Fish seed is critical to the growth of fish farming and aquaculture in general; thus, studies on improving reproductive performance using dietary lipids should be encouraged to increase commercial production of freshwater fish species in aquaculture to meet the global daily demand for fish and fishery products.

## Figures and Tables

**Table 1:** Summary of fish species, lipid sources, and their optimum inclusion levels and their effects on reproductive parameters.

Fish species	Lipid source	Lipid levels	Fecundity	Fertilization rate (%)	GSI (%)	Total egg mass	Larval survival (%)	Hatching rate (%)	Reference
Striped catfish (*Pangasius hypophthalmus*)	VGO, FFO	20 g kg^−1^		80			64	73	[86]
Striped catfish (*Pangasius hypophthalmus*)	FO, CPO	12%	17.18 (eggs·kg^−1^) ×10^4^	80.28	10.48	1.06 (mg)			[20]
P. hypophthalmus X *Pangasius larnaudii*	VGO, FFO	2%			4.42				[18]
Channel catfish (*Ictalurus punctatus*)	MFO, PF	10%	7676.9			678.0 (mL)	91.7	93.9	[96]
Ictalurus punctatus X *Ictalurus furcatus*	MFO, DHA oil, ARA oil	20 g kg^−1^				7266 (mL)		77	[101]
Yellow pike (*Sander vitreus*)	FO, LNO, PNO, SO, PLO	15%		97.7					[93]
Silver catfish (*Rhamdia quelen*)	FO, PO, SO	5%	323.31	78.65				61.29	[87]
Pearl gourami (*Trichogaster leeri*)	KFO, SO	8%	5,556	86	10.75		55.58	75.00	[102]
Nile tilapia (*Oreochromis niloticus*)	CLO, PO	9.7%	823.3	78.5		4385.7 (mL)		60.1	[103]
*Oreochromis karongae*		2-6%	271.3						[82]
Rainbow trout (*Oncorhynchus mykiss*)	PO, FO, CAO,CCO, CO, LNO, OLO, SFO	12.9%	2.2 (eggs × 10^3^ kg^−1^BW)	91.7		92.9 (mg)		89.6	[99]
Zebrafish (*Danio rerio*)	FO	4.1%					99.33	89.33	[51]
Zebrafish (*Danio rerio*)	PC, PE	20%		183			62. 5	93.8	[85]
Common carp (*Cyprinus carpio*)	SFO, FO	3-5%	1.25 (RF × 10^5^)	73.40					[83], [83]
Common carp (*Cyprinus carpio*)	OC	15%	355963.33	84.00	32.08			87.33	[75]
Fancy koi, (*Cyprinus carpio var. koi*)	FO, PO, GNO, VGO	7.5-9.5%	120522	58.33	28.22		26.28	51.38	[88]
*Channa striatus*	SO, FO	18%	32625		11.9		86.0	74.4	[19]
*Anguilla japonica*	PLO, CO	7%		35.1 (%)^1^				9.6 (%)^2^	[74]
*Anguilla anguilla*	FO, RO	5.3-9.2%		69.35				7.81	[12])
*Acipenser ruthenus*	KFO, RO, VGO	3-6%					93.86		[104]
*Acipenser sinensis*	FO	10-18%			Stage III				[80]
*Acipenser baeri*	FO, SOL	13.6%	123.1	86.25		1.73 kg	64.60	81.5	[65]

The values in the table show the studied groups that performed the best overall. Lipid levels presented are the optimum from reported studies. VGO: vegetable oil; FFO: freshwater fish oil; FO: fish oil; CPO: crude palm oil; MFO: menhaden fish oil; PF: poultry fat; LNO: linseed oil; PNO: peanut oil; SO: soybean oil; PLO: pork lard oil; PO: palm oil; KFO: kilka fish oil; CLO: cod liver oil; CAO: canola oil; CCO: coconut oil; OLO: olive oil; SFO: sunflower oil; SEO: sand eel oil; RO: rapeseed oil; OC: oil cake; GNO: groundnut oil; PLO: pollack liver oil; CO: corn oil; SOL: soy lecithin; PC: phosphatidylcholine; PE: phosphatidylethanolamine.

**Table 2:** Summary of lipid metabolism-related genes modulating reproduction in freshwater finfish.

Enzymes/genes/transcriptional factors	Abbreviation	Lipid metabolism function	Dietary lipid source/level	Influence on reproduction	Freshwater fish (species)	Reference
Apolipoprotein	APO	Fatty acid transport	Kilka fish oil, rapeseed oil	Improved egg fertilization, increase larval survival	Sterlet sturgeon (*Acipenser ruthenus*)	[104]
Elongases of very long-chain fatty acids	ELOVL	Fatty acid elongation	Linseed oil, squid oil	Improve spawning success	Swordtail (*Xiphophorus hellerii*); striped snakehead (*Channa striata*)	[10, 111]
Fatty acyl desaturase	FAD	Fatty acid desaturation	Linseed oil, squid oil	Improve spawning success	Swordtail (*Xiphophorus hellerii*); zebrafish (*Danio rerio*); striped snakehead (*Channa striata*)	[10, 111, 112]
Fatty acid binding protein	FABP	Fatty acid transport	18% fish oil and soybean oil mix	Stimulate ovary development to maturation stage IV	Chinese sturgeon (*Acipenser sinensis*)	[68]
Lipoprotein lipase	LPL	Uptake of fatty acid	18% fish oil and soybean oil mix	Stimulate ovary development to maturation stage IV	Chinese sturgeon (*Acipenser sinensis*)	[68]
